# One-step generation of mice carrying a conditional allele together with an HA-tag insertion for the delta opioid receptor

**DOI:** 10.1038/srep44476

**Published:** 2017-03-16

**Authors:** Dongru Su, Min Wang, Chenli Ye, Jiahui Fang, Yanhui Duan, Zhenghong Zhang, Qiuhong Hua, Changjie Shi, Lihong Zhang, Ru Zhang, Xin Xie

**Affiliations:** 1 Shanghai Key Laboratory of Signaling and Disease Research, Laboratory of Receptor-based Bio-medicine, Collaborative Innovation Center for Brain Science, School of Life Sciences and Technology, Tongji University, Shanghai 200092, China; 2 CAS Key Laboratory of Receptor Research, National Center for Drug Screening, Shanghai Institute of Materia Medica, Chinese Academy of Sciences, Shanghai 201203, China

## Abstract

G protein-coupled receptors (GPCRs) are important modulators of many physiological functions and excellent drug targets for many diseases. However, to study the functions of endogenous GPCRs is still a challenging task, partially due to the low expression level of GPCRs and the lack of highly potent and selective GPCR antibodies. Overexpression or knock-in of tagged GPCRs, or knockout of specific GPCRs in mice, are common strategies used to study the *in vivo* functions of these receptors. However, generating separate mice carrying tagged GPCRs or conditional alleles for GPCRs is labor intensive, and requires additional breeding costs. Here we report the generation of mice carrying an HA-tagged DOR (delta opioid receptor) flanked by LoxP sequences at the endogenous DOR locus using a single recombination step, aided by the TALEN system. These animals can be used directly to study the expression, localization, protein-protein interaction and signal transduction of endogenous DOR using anti-HA antibodies. By crossing with mice expressing tissue-specific Cre, these mice can also generate offspring with DOR knockout within specific tissues. These mice are powerful tools to study the *in vivo* functions of DOR. Furthermore, the gene modification strategy could also be used to study the functions of many other GPCRs.

G protein-coupled receptors (GPCRs), also called seven-transmembrane receptors, form the largest, most versatile and most ubiquitous membrane receptor family^1^. These receptors can be activated by a variety of ligands ranging from light, ions, to small molecule neurotransmitters and peptide hormones, and modulate virtually all known physiological processes^2^. They are also excellent drug targets, nearly 36% of drugs on the market target the GPCRs, either directly or indirectly^3^. In recent years, the determination of the crystal structure of many GPCRs has provided us with insights into GPCR-ligand interaction and the structural basis of GPCR activation at the atomic level^2^,^4^,^5^. However, to study the function of endogenous GPCRs is still a challenging task, partially due to the low expression level of GPCRs and the lack of highly potent and selective GPCR antibodies^6^,^7^,^8^. Delta opioid receptor (DOR) is a GPCR which plays important roles in analgesia^9^,^10^, anxiety^11^, substance abuse^12^, neuro-protection^13^,^14^, cardiac protection^15^ and immune response^16^,^17^. The studies of DOR also suffer from a lack of specific antibodies^9^,^18^,^19^. Scientists have claimed that many commonly used anti-DOR antibodies do not recognize the DOR in immunohistochemical preparations, but rather cross-react with an unidentified molecule^18^.

Mice with specific GPCR knockout are widely used to study the function of the receptors *in vivo*^20^,^21^. A number of reports also generated mice with reporter genes, such as GFP, luciferase or β-galactosidase, inserted after the promoter of certain GPCRs, to monitor the expression of endogenous receptors^22^,^23^,^24^. However, apart from reporting the expression pattern, these reporters cannot help the study of receptor function, such as protein-protein interactions and signal transduction. In *in vitro* studies, overexpression of tag-fused GPCRs in cell lines is a commonly adopted approach used to study the functions of GPCRs^25^,^26^. Generating transgenic mice with overexpression of tag-fused GPCRs is a simple way to mimic the *in vitro* study. However, overexpression of GPCR may lead to deviations in its original function^27^. The precise knockin of a tag-fused GPCR at its endogenous position in the genome of mice would provide us an ideal tool, thus avoiding the unpredictable consequences of receptor overexpression. Meanwhile, if LoxP sequences^28^,^29^,^30^ could be added flanking the tagged GPCR, these mice could be used to generate offspring with tissue-specific or time-specific knockout of this GPCR by crossing with mice expressing tissue specific or inducible Cre recombinase.

Recent advances in gene-editing technology such as the use of zinc finger nucleases, TALEN and CRISPR/Cas9^31^,^32^ provide us with new ways of precise insertion of sequences into target genes. TALEN stands for transcription activator-like effector nucleases and are engineered restriction enzymes which contain a TAL effector DNA-binding domain recognizing a specific DNA sequence and a DNA cleavage nuclease domain. By inducing a double strand DNA break at a specific location, this enzyme facilitates homologous recombination and allows the insertion of designed sequences at the targeted location^33^. Here, we report the one step generation of mice carrying an HA-tag insertion and a conditional allele for DOR by using TALEN. These mice express HA-DOR N-terminal fusion in place of the native DOR. Meanwhile, DOR can be knocked-out within defined tissues after crossing with mice expressing tissue-specific Cre recombinase. This mouse would be an excellent tool to study the expression, distribution and function of the DOR gene *in vivo*.

## Results

### Design of TALEN and HA-DOR donor plasmid

**Figure 1:**
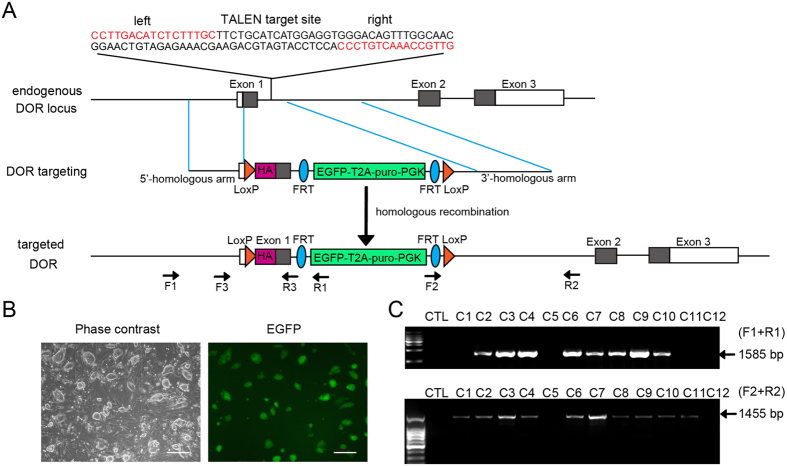
TALEN design and targeting of DOR gene in mouse embryonic stem cells (mESCs). (**A**) Schematic representation of the DOR gene, DOR targeting construct (donor plasmid) and genotyping strategy. The TALEN-recognition sequences are shown in red. The coding sequence of DOR gene is represented by gray box. The LoxP recombination sites flanking the ORF within exon 1 are labeled as orange triangles. HA-tag (magenta box) is located within exon 1 to express an HA-DOR N-terminal fusion in place of the native DOR. The targeting construct also contains a puromycin (puro) and EGFP cassette flanked by FRT recombination sites. F1, R1, F2, R2, F3, R3 were primers used for PCR analysis. (**B**) Representative image of the EGFP positive mESCs clone (C4). The left panel is the phase contrast view of the right. Scale bars represent 100 μm. (**C**) PCR analysis of homologous recombination at the DOR locus of EGFP positive mESC colonies (C1-C12) that were cotransfected with donor plasmid and TALEN pair plasmids. CTL, negative control.

The DOR gene has only one transcription product and the coding region of DOR starts within exon 1 (Fig. 1A and supplementary info). If the TALEN-recognition sequence is located in the ORF (open reading frame) of DOR, the TALEN nuclease may keep on cutting the target site even after homologous recombination since the HA-DOR donor plasmid also contains these sequences. This may lead to unexpected mutation of the target gene. So we selected a TALEN-recognition sequence within intron 1 (Fig. 1A and supplementary info, Mus musculus chromosome 4: 132143894-132143943), and this sequence would be replaced after successful recombination. The repeat array in the TALEN pair responsible for DNA recognition was designed by matching individual repeat variable diresidues (RVD) to specific DNA bases and was assembled in the TALEN pair plasmids harbouring a CAG promoter (details in the Methods).

We then designed a DOR donor plasmid for the murine DOR gene with 886 bp 5′-homologous arm (Mus musculus chromosome 4: 132145281-132144396) and 1181 bp 3′-homologous arm (Mus musculus chromosome 4: 132143909-132142729). An HA-tag sequence was inserted right before the ORF of DOR gene to allow the expression of an HA-DOR N-terminal fusion. The LoxP recombination sites were inserted after the 5′-homologous arm and before the 3′-homologous arm (Fig. 1A and supplementary info). The plasmid also contained EGFP and a puromycin resistance cassette flanked by FRT recombination sites (EGFP-T2A-puro-PGK, ETPP), which could be used to screen cells or mice in which the homologous recombination at the DOR locus occurs (Fig. 1A and supplementary info). The length of the DNA fragment between the 5′ homologous arm and 3′ homologous arm is 2918 bp.

### Targeting of DOR gene in mouse embryonic stem cells (mESCs)

To test the efficiency and accuracy of TALEN-aided homologous recombination, mESCs were co-transfected with TALEN pair plasmids and DOR donor plasmid. Twenty-four hours after transfection, mESCs were supplemented with fresh medium containing 0.5 μg/ml puromycin. Six days later, most of the surviving mESC colonies were positive for EGFP, indicating the incorporation of donor sequence (Fig. 1B). Twelve EGFP positive colonies were selected for PCR analysis (Fig. 1C). The targeting events were identified by two sets of primers (Fig. 1A and supplementary info). The correct PCR product using primers F1 (within the DOR gene locus) and R1 (within the donor sequence) demonstrates the correct insertion of the donor sequence at the 5′ end, while the correct PCR product using primers F2 (within the donor sequence) and R2 (within the DOR gene locus) ensures the correct ligation at the 3′ end. The PCR results showed that both products, one is 1585 bp in length and the other 1455 bp in length, were obtained in eight out of the 12 colonies (Fig. 1C, C2-4, C6-10), indicating correct insertion of the donor sequence into the DOR locus in these mESCs. The successful rate of precise homologous recombination was 67%. Therefore, these TALEN pair plasmids and DOR donor plasmid were used in subsequent experiments.

### Generation of mice carrying HA-tag and conditional allele for DOR

**Figure 2:**
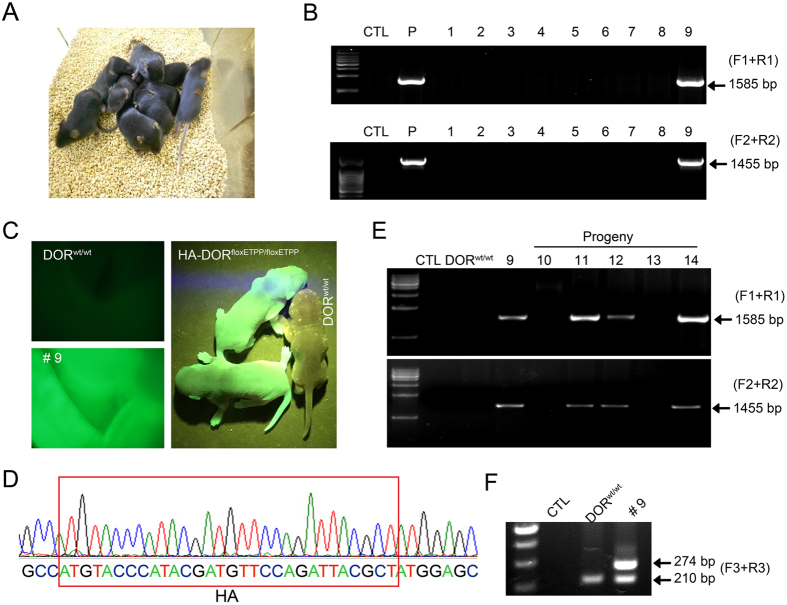
Generation of mice carrying an HA-tag and a conditional allele for DOR. A combination of TALEN mRNA (20 ng/μl, 1–2 pl/oocyte) and linearized DOR donor plasmid (20 ng/μl, 1–2 pl/oocyte) were injected into fertilized oocytes. After injection, the surviving zygotes were transplanted into foster mothers and postnatal mice were obtained and genotyped. (**A**) The picture of nine mice which were born after microinjection (No. 1–9). (**B**) PCR genotyping of the nine mice with F1, R1, F2, R2 primers. P, positive control (EGFP positive mESCs clone: C4). (**C**) Fluorescence image of the abdomen of the DOR^wt/wt^ (left top) and HA-DOR^floxETPP/wt^ (ETPP, EGFP-T2A-puro-PKG) mouse (No. 9, left bottom) and the F5 progeny of No. 9 mouse (right). (**D**) Nucleotide sequencing demonstrated HA insertion (red rectangles) in the correct location in the HA-DOR^floxETPP/wt^ mouse (No. 9). (**E**) PCR genotyping of the F1 progeny (No. 10–14) of mouse No. 9. (**F**) PCR analysis of LoxP and HA insertion with F3, R3 primers in DOR^wt/wt^ and No. 9 mouse.

To facilitate the expression of TALEN proteins in zygotes, the TALEN pair plasmids were linearized and mRNA was transcribed *in vitro*. Then TALEN mRNA (20 ng/μl, 1–2 pl/oocyte) and DOR donor plasmid (20 ng/μl, 1–2 pl/oocyte) were injected into fertilized mouse oocytes. Seventy-two out of the 100 fertilized oocytes survived the injection. They were transferred into the oviducts of pseudo-pregnant fosters mothers, and gave rise to ten newborns (one died soon after birth) (Fig. 2A). Tail genomic DNA of those 9 surviving newborns was used for genotyping. Luckily we found that founder mouse No. 9 were positive for both 1585 bp and 1455 bp PCR products, described above, which suggested the precise homologous recombination at the DOR locus (Fig. 2B). Furthermore, compared to wild type mice (DOR^wt/wt^), mouse No. 9 was EGFP positive. Nucleotide sequencing of mouse No. 9 demonstrated an HA insertion in the correct location (Fig. 2D) as well as the precise insertion of LoxP, FRT, puromycin and EGFP sequences. The inserted sequence exactly matched the sequence in the donor plasmid between the 5′- and 3′-homologous arms (supplementary info).

To test whether the DOR knock-in allele could be transmitted through the germline, mouse No. 9 (female) was crossed with wild type C57BL/6 mice and the genotypes of the five F1 progeny (No. 10–14) were determined by PCR analysis. Three out of the five F1 progeny (No. 11, 12 and 14) were positive for both the 1585 bp and 1455 bp PCR products (Fig. 2E), and all mice carrying the HA-DOR^floxETPP^ allele among the F5 progeny of mouse No. 9 were positive for EGFP (Fig. 2C), demonstrating successful germline transmission. According to Mendelian inheritance, we could deduce that only one allele of the DOR gene was modified by homologous recombination in founder mouse No. 9, and this deduction was further confirmed by PCR analysis with another pair of primers F3 and R3 (Fig. 1A). Both the 210 bp and 274 bp PCR products were detected in founder mouse No. 9; while in wild type mice, only the 210 bp PCR product was detectable (Fig. 2F). These mice carrying one copy of the donor sequence were named HA-DOR^floxETPP/wt^.

### Conditional disruption of mouse DOR gene

**Figure 3:**
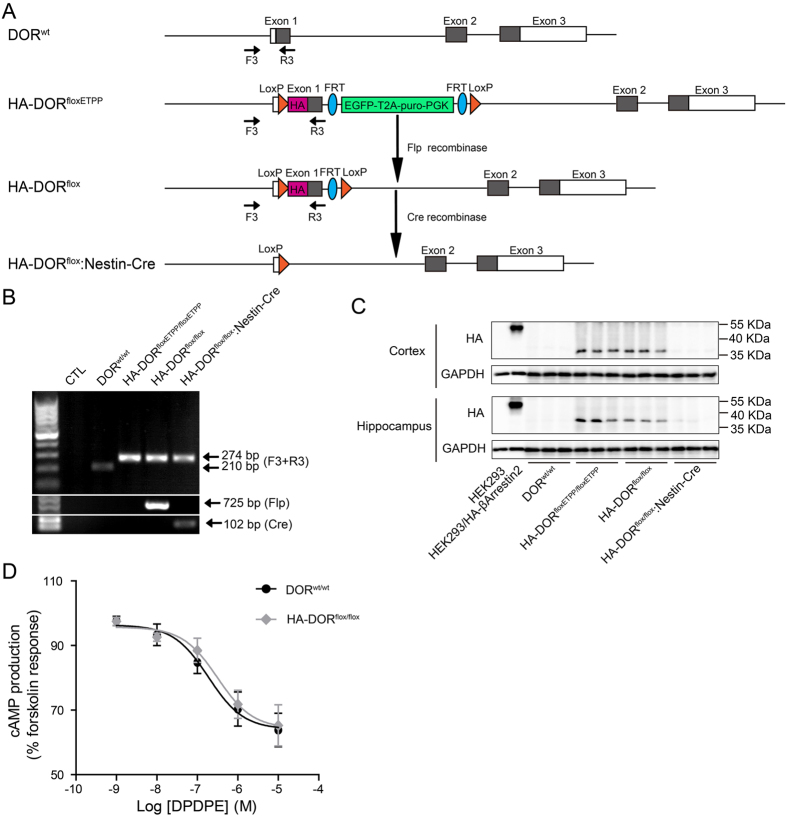
Flp and Nestin-Cre mediated gene excision *in vivo*. (**A**) Schematic of Flp- and Cre-mediated gene excision. F3, R3 were primers used for PCR genotyping. (**B**) PCR analysis of Flp- and Nestin-Cre-mediated gene excision in DOR^floxETPP/floxETPP^ mice. Using tail genomic DNA, the 210 bp PCR product was only detected in DOR^wt/wt^ mouse, and the 274 bp PCR product were detected in HA-DOR^floxETPP/floxETPP^, HA-DOR^flox/flox^, HA-DOR^flox/flox^:Nestin-Cre mice. Using Flp primers, the 725 bp PCR product was only detected in HA-DOR^flox/flox^ mouse, which was generated by crossing HA-DOR^floxETPP/floxETPP^ with Rosa26-Flp mouse. Using Cre primers, the 102 bp PCR product was only detected in the progeny of HA-DOR^flox/flox^ and Nestin-Cre transgenic mouse (HA-DOR^flox/flox^:Nestin-Cre). (**C**) Western blot analysis of HA-DOR expression in the cortex and hippocampus from DOR^wt/wt^, HA-DOR^floxETPP/floxETPP^, HA-DOR^flox/flox^ and HA-DOR^flox/flox^:Nestin-Cre mice by using anti-HA antibody (n = 3). HEK293 cells overexpressing HA-βArrestin2 were used as a positive control. GAPDH was used as a loading control. (**D**) Dose-dependent inhibition of forskolin-induced cAMP production in cortical neurons isolated from DOR^wt/wt^ and HA-DOR^flox/flox^ mice.

To delete the EGFP and puromycin cassette flanked by the FRT recombination sites, the heterozygous HA-DOR^floxETPP/wt^ mice were crossed with Rosa26-Flp mice to generate HA-DOR^flox/wt^ mice (Fig. 3A). Then the HA-DOR^flox/wt^ mice were crossed with each other to obtain homozygous HA-DOR^flox/flox^ mice, which could be used to study DOR with anti-HA antibodies, or used to generate conditional DOR knockout mice by crossing with mice with tissue specific Cre expression. Since DOR is most highly expressed in neurons, the HA-DOR^flox/flox^ mice were crossed with Nestin-Cre mice to generate mice with neural-specific DOR knockout (HA-DOR^flox/flox^:Nestin-Cre, Fig. 3A).

Tail genomic DNA of those mice was used for PCR genotyping. Using F3 and R3 primers (Fig. 3A), the 210 bp PCR product was only detected in DOR^wt/wt^ mice, and the 274 bp PCR product was detected in HA-DOR^floxETPP/floxETPP^, HA-DOR^flox/flox^, HA-DOR^flox/flox^:Nestin-Cre mice (Fig. 3B). Since Nestin-Cre is only expressed in the central nervous system, it’s reasonable that the 274 bp PCR product was still detectable in the tail genomic DNA of the HA-DOR^flox/flox^:Nestin-Cre mice. Using Flp recombinase specific primers, the 725 bp PCR product indicative of the presence of the Flp transgene, was only detected in HA-DOR^flox/flox^ mice, which were progeny generated with Rosa26-Flp mice. Using Cre recombinase specific primers, the 102 bp PCR product, indicative of the presence of the Cre transgene, was only detected in HA-DOR^flox/flox^:Nestin-Cre mice.

In order to prove the correct insertion and function of HA-tag, and the ablation of DOR in the central nervous system, western blot analysis were performed. DOR is mainly expressed in brain and spinal cord, so we detected the expression of HA-DOR in cortex and hippocampus with an anti-HA antibody (Fig. 3C). HEK 293 cells transfected with plasmids encoding HA-βArrestin2 were used as a positive control of the anti-HA antibody. As expected, no HA signal could be detected from wild-type mice, and a clear HA-positive signal was detected from the cortex and hippocampus lysates of HA-DOR^floxETPP/floxETPP^ and HA-DOR^flox/flox^ mice. Furthermore, after tissue-specific deletion of the HA-DOR, the HA-signal disappeared in the brain samples of HA-DOR^flox/flox^:Nestin-Cre mice.

Next, we explored whether the insertion of LoxP and HA sequences influence the function of DOR. Cortical neurons from DOR^wt/wt^ or HA-DOR^flox/flox^ mice were isolated and cultured *in vitro* for 15 days. These neurons were stimulated with various concentrations of DOR agonist DPDPE, followed by adenylate cyclase activator forskolin (2.5 μM). DPDPE was found to dose-dependently inhibit forskolin-stimulated cAMP production from these neurons. And DPDPE displayed almost identical potency and maximal response in DOR^wt/wt^ and HA-DOR^flox/flox^ neurons (Fig. 3D), indicating normal function of DOR in the HA-DOR^flox/flox^ mice.

**Figure 4:**
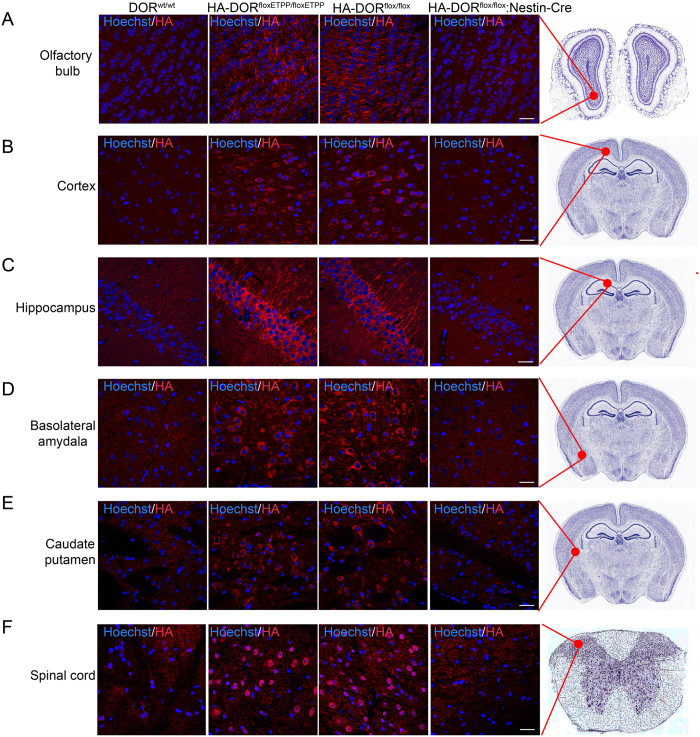
Detection of HA-DOR in brain sections with immunofluorescent staining. Brain sections from DOR^wt/wt^, HA-DOR^floxETPP/floxETPP^, HA-DOR^flox/flox^ and HA-DOR^flox/flox^:Nestin-Cre mice were stained with anti-HA antibody and fluorescence-conjugated secondary antibody (red). Cell nuclei were labeled with Hoechst 33342 (blue). Representative confocal images were shown. (**A**) olfactory bulb, (**B**) layer V (internal pyramidal layer) of the cortex, (**C**) pyramidal cell layer of the hippocampal region CA1, (**D**) basolateral amydala, (**E**) caudate putamen and (**F**) spinal cord. Scale bars represent 20 μm.

We also examined the distribution of HA-DOR in mouse brain with immunofluorescent staining using anti-HA antibody (Fig. 4). For HA-DOR^floxETPP/floxETPP^ and HA-DOR^flox/flox^ mice, strong HA-DOR expression could be detected in olfactory bulb, layer V (internal pyramidal layer) of the cortex, pyramidal cell layer of the hippocampal region CA1, basolateral amydala, caudate putamen and spinal cord. Meanwhile, we couldn’t detect specific HA signal in the DOR^wt/wt^ or HA-DOR^flox/flox^:Nestin-Cre mice. In conclusion, we have successfully generated mice carrying HA-tag and conditional allele for DOR, which is an excellent tool for studying the function of DOR *in vivo*.

## Discussion

Antibodies are essential tools for functional analysis of endogenous proteins. Unfortunately, high affinity GPCR antibodies are very difficult to generate due to: (1) lack of a suitable antigen and a synthetic linear GPCR may not represent the structural feature of a native GPCR^7^; (2) low expression of GPCR on the cell surface^8^; (3) the subtypes of GPCR typically have high degrees of homology and hence antibodies may recognize other subtypes within the same family^6^. Consequently, the functions of many GPCRs have been studied in cell-based assays with overexpression of Tag-fused GPCRs^34^,^35^,^36^, as these genetically modified HA-, Flag-, His-, or (E)GFP-tagged receptors, can easily be detected or immunoprecipitated using antibodies against the respective tag.

A number of studies also generated transgenic mice with overexpression of tag-fused GPCRs to study the *in vivo* functions of GPCRs^37^,^38^. However, overexpression of GPCR may lead to deviation from its true functions^27^. So to precisely knock-in a tag-fused GPCR at its endogenous position in the genome of mice would provide us with an ideal tool. Using GPR49-EGFP-IRES-CreERT2 mice^39^, GPR49 was found to be marker of normal tissue stem cells in small intestine and the unique ultrastructural anatomy of GPR49^+^ cycling crypt base columnar cells was also revealed. Although a reliable OPRL antibody is still lacking, detailed OPRL expression in brain, spinal cord and dorsal root ganglion neurons was revealed by using the OPRL-EGFP mice^40^. Scherrer *et al* replaced DOR with an active DOR-EGFP fusion to produce DOR-EGFP knock-in mice that allowed visualization of DOR *in vivo* and revealed the distribution of DOR in mouse brain^22^. With DOR-EGFP/MOR (Mu Opioid Receptors)-mCherry double knock-in mice, Erbs and colleagues generated an online interactive database offering concomitant MOR/DOR visualization at subcellular resolution and studied the potential interactions between the two pathways^41^.

On the other hand, knockout of specific genes in mice is also a widely accepted way to study *in vivo* functions of genes. The endogenous functions of many GPCRs have been discovered via the gene deletion strategy^42^,^43^. To study tissue-specific or time-dependent functions, a number of recombinase systems, including the widely used Cre/LoxP system, have been developed to generate tissue-specific knockout mice^44^. Many GPCR genes have been modified to carry LoxP sequences and tissue-specific functions have been identified^45^,^46^,^47^. Chung *et al* generated a conditional DOR knockout mouse line and ablated the DOR gene specifically in GABAergic neurons of the forebrain, revealing that the DOR agonist SNC80 induced epileptic seizures via direct inhibition of GABAergic forebrain neurons^48^.

However, generating separate mice carrying tag-GPCR or conditional allele for GPCR is labor intensive, and would incur significant additional costs for both the generation and the housing of two independent models. In addition, although the DOR-EGFP knockin mice^22^ are useful in visualizing the *in vivo* localization of DOR, the C-terminal EGFP tag cannot be used to specifically immunoprecipitate plasma membrane-localized DOR since it is located at the intracellular side of the 7 transmembrane DOR. Cells have to be permeabilized for the antibodies to access the C-terminal tags, and this would result in the total receptor content of the cell in all the subcellular compartments being pulled down together. Since plasma membrane is the main site of action for most GPCRs, including DOR, it is important to study separately DOR localized on the cell surface or in intracellular compartments. An N-terminal (extracellular) HA-tag would be more useful in identifying only the plasma membrane-localized DOR since antibodies can directly bind to the HA-tag without damaging the cell membrane. It would be interesting to compare the endogenous DOR interacting proteins immunoprecipitated with antibodies against N-terminal tags (representing membrane-localized DOR) and C-terminal tags (representing total DOR). The HA-DOR can also internalize with the binding antibodies after ligand stimulation, and this makes it possible to trace the trafficking of those membrane-localized receptors. For this extra flexibility, we have designed and generated these “two-in-one” mice with both an N-terminal HA-tag and with LoxP sites inserted into specific locations in the DOR gene to facilitate conditional ablation of the gene.

The successful replacement of the endogenous DOR gene by the HA-DOR flanked by LoxP sequences benefited from the recent advances in gene-editing techniques. In this study we used the TALEN system to facilitate the homologous recombination by inducing a double strand break at a specific site with the intron of the DOR gene. In mESCs, the recombination efficiency reached 67%. In mice, one out of nine surviving pups carrying the correct gene insertions, representing a 11.1% recombination efficiency. These results have again demonstrated that the TALEN system is an efficient tool for the generation of genetically modified animal models. Recently, the clustered regularly interspaced short palindromic repeats (CRISPR)/Cas9 system has been reported to be a more efficient and simple tool for genome editing. However, the high frequency of off-target activity associated with the CRISPR/Cas9 system is a major concern^49^. The specificity of TALEN is determined by the combination of two TALEN DNA binding domains, because TALENs only function when dimers between the FokI nuclease domains come together. The specificity of CRISPR/Cas9 system is only determined by the presence of the PAM sequence and the 20 nucleotides upstream of the PAM site in the target genome^50^. Various strategies have been reported to optimize CRISPR/Cas9 system and reduce off-target effect^51^.

In conclusion, mice carrying an HA-tag and a conditional allele for DOR were generated with one recombination step aided by the TALEN system. These animals can be used directly to study the expression, localization, protein-protein interaction and signal transduction of endogenous DOR. By crossing with mice expressing tissue-specific Cre, these mice could also generate offspring with DOR knockout within specific tissues. These mice are a useful tool for the study of the *in vivo* functions of DOR. The gene modification strategy adopted in this study could also be used to investigate the functions of many other GPCRs.

## Methods

### Animals

C57BL/6 mice were obtained from Shanghai Laboratory Animal Center (Chinese Academy of Sciences). Rosa26-Flp mice (Jackson Laboratory, stock number 003946) harbor the Flp knock-in allele with widespread expression of the Flp recombinase driven by the *Gt(ROSA)26Sor* promoter. Nestin-Cre transgenic mice (Jackson Laboratory, stock number 003771) express Cre recombinase under the control of a rat Nestin promoter. All experiment procedures for the use and the care of the animals complied with international guidelines for the care and use of laboratory animals and were approved by the Animal Ethics Committee of Tongji University, Shanghai, China.

### Generation of TALEN pair plasmids and DOR donor plasmid

TALEN pair plasmids (pCS2-peas/perr-T) were purchased from ViewSolid Biotech (Beijing, China). The TALEN recognition site for *DOR* gene is located on Mus musculus chromosome 4: 132143894-132143943, and the repeat array in the TALEN pair responsible for DNA recognition was designed by matching individual repeat variable diresidue (RVD) to specific DNA bases. The sequences of repeat array were synthesized by ViewSolid Biotech (Beijing, China) and assembled in the NheI site of pCS2-peas/perr-T plasmids which encode a CAG promoter-driven TALEN scaffold. The DOR donor plasmid was generated with a backbone plasmid containing an FRT-flanked PGK-puromycin-T2A-EGFP cassette and a downstream LoxP site (ViewSolid Biotech, Beijing, China, supplementary note). Firstly, a 1348 bp fragment (Mus musculus chromosome 4: 132145281-132143933) spanning the 5′-homologous arm (Mus musculus chromosome 4: 132145281-132144396), exon 1 and part of intron1 was amplified by PCR using 5′ arm F/R primers and ligated into a shuttle vector (pEGFP-N1). The 5′ LoxP site was inserted into the downstream of the 5′-homologous arm by inverse PCR using LoxP F/R primers (KOD plus mutagenesis kit, Toyobo life science). HA-tag was inserted right before the open reading frame (ORF) of DOR gene by another round inverse PCR using HA F/R primers. 1181 bp 3′-homologous arm (Mus musculus chromosome 4: 132143909-132142729) was amplified with 3′ arm F/R primers. Subsequently, the 5′ arm-LoxP-HA and 3′ arm were inserted into the corresponding multiple cloning sites of the donor backbone plasmid to obtain the final DOR donor plasmid (primers listed in Table S1).

### Transcription of TALEN mRNA *in vitro*

For DOR gene targeting in fertilized oocytes, TALEN pair plasmids were linearized with NotI and TALEN mRNA was transcribed using mMESSAGE mMACHINE SP6 Kit (Ambion) following the manufacturer protocol. TALEN mRNA was stored at −80 °C until use (not more than 6 weeks).

### Cell culture and selection

mESCs were cultured in DMEM (Gibco) supplied with 10% FBS (Hyclone), 0.1 mM non-essential amino acids (NEAA; Gibco), 2 mM glutamax (Gibco), 0.1 mM 2-mercaptoethanol (Gibco), 100 units/ml penicillin and 100 μg/ml streptomycin (Gibco) at 37 °C in 5% CO_2_. mESCs were plated on feeder layers and passaged with 0.25% trypsin/0.02% EDTA.

mESCs were seeded onto six-well plates at a density of 500 000 cells per well. At 50–60% confluency, mESCs were transfected with 1 μg linearized TALEN pair plasmids (digested with NotI) and 1 μg linearized DOR donor plasmid (digested with XmnI) by using Lipofectamine 2000. 24 h after transfection, the medium was replaced with fresh medium supplied with 0.5 μg/ml puromycin (InvivoGen). Six days later, EGFP positive colonies were collected for PCR genotyping.

### Microinjection of fertilized oocytes

Prepubescent C57BL/6 females (4–6 weeks old) were injected with 7.5–10 IU pregnant mare serum gonadotropin (Ningbo Sansheng Pharmaceutical Company, China). 48 h later, they were injected with 7.5–10 IU human chorionic gonadotropin (Ningbo Sansheng Pharmaceutical Company, China). After breeding, fertilized oocytes were collected for subsequent microinjection. A mixture of TALEN mRNA (20 ng/μl) and linearized DOR donor plasmid (20 ng/μl) was prepared by dilution in microinjection buffer (10 mM Tris, 0.1 mM EDTA, pH 7.4). Subsequently, 1–2 pl of the mixture was injected into each fertilized oocyte by using a microinjector. The surviving zygotes were transferred into the oviducts of pseudopregnant fosters mothers.

### Genotyping experiments

EGFP positive and puromycin-resistant mESCs colonies were collected for PCR genotyping. Genomic DNA was extracted from colonies by using Blood and Tissue DNA Mini Kit (Aidlab Biotechnologies, China). For genotyping of mice, tail genomic DNA was extracted by using Mouse Direct PCR kit (Biotool). Then, the DNA lysates were subjected to PCR analysis with primers listed in Table S1.

### Western blot analysis

Cells or brains were lysed in sample buffer [50 mM Tris-HCl, 1% w/v SDS, 1% 2-mercaptoethanol, 15% glycerol, 0.01% bromophenyl blue, protease inhibitor (Roche), phosphatase inhibitor (Sigma), pH 6.8]. Then, the lysates were separated on SDS-PAGE and transferred to PVDF membranes. Membranes were incubated in blocking buffer (5% nonfat milk, 0.1% tween 20 in TBS) for 1 h at room temperature and followed by incubating overnight at 4 °C with rabbit anti-HA antibody (Santa Cruz) and rabbit anti-GAPDH antibody (Cell Signaling Technology). After washing 3 times, membranes were incubated for 1 h at room temperature with HRP-conjugated goat anti-rabbit IgG (Promega) and detected with Western Lightning Ultra (PerkinElmer).

### Isolation of cortical neurons

Cortical tissues were isolated from postnatal (P0-P2) HA-DOR^flox/flox^ or DOR^wt/wt^ mice and incubated in 0.125% trypsin solution for 10 min at 37 °C. After thorough washing, the dissociated cortical cells were seeded at a density of 4 × 10^5^ cells/well onto poly-D-lysine coated 24-well plate in plating medium (DMEM with 10% FBS). Four hours later, the medium was replaced with pre-warmed maintenance medium (Neurobasal medium with B27 supplement and Glutamax I) and cultured at 37 °C. In the first 2 days, cytosine arabinose (1 μM) was added to remove contaminating glia cells and half the medium was changed every 2 days.

### HTRF cAMP Assay

HTRF cAMP dynamic 2 assay kits (Cisbio), which are based on a competitive immunoassay using cryptate-labeled anti-cAMP antibody and d2-labeled cAMP (a fluorescent conjugated cAMP), were used to detect intracellular cAMP. Experiments were performed according to the manufacturer’s instruction 15 days after the plating of cortical neurons. Cells were washed and incubated in phosphate-buffered saline containing various concentration of DPDPE and the phosphodiesterase inhibitor IBMX (500 μM) for 10 min at room temperature, then adenylate cyclase activator forskolin (2.5 μM) was added and incubation continued for 30 min. Lysis buffer containing d2-cAMP and anti-cAMP antibody was then added. After 60-minute incubation at room temperature in the dark, HTRF signal was detected with a FlexStation 3 microplate reader (Molecular Devices, Silicon Valley, CA, USA). The cAMP concentration was calculated according to the fluorescence ratio (665 nm/620 nm).

### Immunostaining

After general anesthesia, mice were transcardially perfused with saline. Brains were fixed in 4% PFA at 4 °C for 24 h and cryoprotected in 30% sucrose for another 24 h. Next, brains were cryosectioned at 30 μm. Brain sections were rinsed in PBS for 3 times, blocked with 2% goat serum and 0.3% Triton X-100 in PBS for 1 h at room temperature. Subsequently, sections were incubated overnight at 4 °C with rabbit anti-HA antibody. After PBS washing, sections were incubated with Alexa Fluor 488 goat anti-rabbit IgG (Invitrogen) and Hoechst 33342 (Sigma) for 1 h at room temperature. Then sections were examined with a confocal microscope.

## Additional Information

**How to cite this article**: Su, D. *et al*. One-step generation of mice carrying a conditional allele together with an HA-tag insertion for the delta opioid receptor. *Sci. Rep.* **7**, 44476; doi: 10.1038/srep44476 (2017).

**Publisher's note:** Springer Nature remains neutral with regard to jurisdictional claims in published maps and institutional affiliations.

## Supplementary Material

Supplementary Information
