# Supplementary material for: One-step generation of mice carrying a conditional allele together with an HA-tag insertion for the delta opioid receptor
Source: Sci Rep. 2017 Mar 16;7:44476. doi: 10.1038/srep44476 (PMC5353682; doi:10.1038/srep44476)
Supplement: Supplementary Information [file srep44476-s1.pdf]

Supplementary information

One-step generation of mice carrying a conditional allele together with an HA-tag insertion for the delta opioid receptor

Dongru Su[1], Min Wang[2], Chenli Ye[1], Jiahui Fang[1], Yanhui Duan[1], Zhenghong Zhang[1], Qiuhong Hua[1], Changjie Shi[1], Lihong Zhang[1], Ru Zhang[1,*], Xin Xie[1,2,*]

[1]Shanghai Key Laboratory of Signaling and Disease Research, Laboratory of Receptor-based Bio-medicine, Collaborative Innovation Center for Brain Science, School of Life Sciences and Technology, Tongji University, Shanghai 200092, China

[2]CAS Key Laboratory of Receptor Research, National Center for Drug Screening, Shanghai Institute of Materia Medica, Chinese Academy of Sciences, Shanghai 201203, China

*Address correspondence to: Dr. Xin Xie, 189 Guo Shou Jing Road, Shanghai 201203, China; Fax: 0086-21-50800721; E-mail: xxie@simm.ac.cn; or Dr. Ru Zhang, 1239 Siping Road, School of Life Sciences and Technology, Tongji University, Shanghai 200092, China, Tel: 86-21-65986852; E-mail: ru.zhang@tongji.edu.cn

Table S1. List of primers used in the experiments.

5' arm F	5'-GGAATTCAATGAGCCAGACAGGAAAGT-3'	5'-homologous arm PCR
5' arm R	5'-GGGGTACCAGATGTCAAGGATGATTAAGTGT-3'	
3' arm F	5'-CCCAAGCTTGGGACAGTTTGGCAACACTT-3'	3'-homologous arm PCR
3' arm R	5'-ATAAGAATGCGGCCGCCTGAACCCCTAATCTGAGCA-3'	
LoxP F	5'-ATAACTTCGTATAGCATACATTATACGAAGTTATCGGCGGCGCCATGGAGCTGGTGCCCT-3'	Inverse PCR
LoxP R	5'-TGTCCGTCTCCACCGTGCGCCCTGG-3'	
HA F	5'-ATGTACCCATACGATGTTCCAGATTACGCTATGGAGCTGGTGCCCTCTGCCCGTGCG-3'	
HA R	5'-GGCGCCGCCGATAACTTCGTATAATGT-3'	
F1	5'-AACAGCACAACTGGTCCATAAGG-3'	5' junction PCR
R1	5'-CCTCCTCCTCGACTAAGCATACC-3'	
F2	5'-GCCCTGTTGTCTGTAGTTTCCA-3'	3' junction PCR
R2	5'-CTGAGCCCATGTTCTAGTCTATGAA-3'	
F3	5'-CGCACGGTGGAGACGGACA-3'	HA insertion PCR
R3	5'-CGCACACAGCCGAGTAGAGC-3'	
Flp F	5'-CACTGATATTGTAAGTAGTTTGC-3'	Flp genotyping
Flp R	5'-CTAGTGCGAAGTAGTGATCAGG-3'	
Cre F	5'-GCGGTCTGGCAGTAAAAACTATC-3'	Cre genotyping
Cre R	5'-GTGAAACAGCATTGCTGTCACTT-3'	

Supplementary information

Sequence of DOR donor plasmid:

1. Sequences of DOR gene locus including exon 1 and part of the first intron are in red (Mus musculus chromosome 4: 132145281-132143933 and 132143909-132142729).

2. Sequences within **[ ]** represent the 5'-homologous arm, sequences within **( )** represent the 3'-homologous arm.

3. Red bold sequences are the coding region within exon 1 of the DOR gene.

4. HA-tag sequences are in blue and LoxP sites are in green.

5. The underlined sequences are the FRT-flanked pGK-puromycin-T2A-EGFP cassette in the donor plamid (FRT, EGFP, T2A, Puromycin, PGK promoter).

gaaccagatctgatatcatcgatgaatt**[**caatgagccagacaggaaagttgtgtct
ctaataacatacacacatctgtccagataggtgtgaccttttcagcttgatgtagt
gagatggttagtgaccttgattgcggcttatgtctgccatgtccggtgccaagctgt
acagacccaggtgggcttatttccagtgtctccccttgaggttgtagctgatcatat
tactagttttcatccggagcctttggggaactggacggttttgtttttgtttcttg
gtcaggaatcacttggcagttctagaagacacggcaaggagcagagcgcacacagta
cacggggtggtggtggtggtggtggtggtggtggtggtggtggaaatctgagt
tctttactccgttgtcttcatctccccaaaccctctgaaggcggcaggagatttc
ttcatcgtgcagtcacagagaatgaacttgagagtgcttaagcggctcgctcaaggt
tactcccatcgtccgtgaagaggcgggaggcgggattcgcatccaggttcttctgac
tccgaagtggcacggggtggggcgggaggctgggccgggctgggggcacctggagt
cagcggagcccaggcagggagcgcgcgcgaggcctctctggcccagctcggagcccg
gtggtgcagcgcccgcccccgatcgcgggcgcagactggcggccgcctccggaccac
gtggtgcgcgcggcggctcggggctccccgggcttgggcgagcactgcgggcggtcg
accgggcggaggaggcgggagcgagcgctgcagcctgcggcgtcggggccgtggcct
ccgttttccgcgcgccactcgctgggtccctgcgcccagggcgcacggtggagacgg
aca**]**ataacttcgtatagcatacattatacgaagttatcggcggcgccatgtaccca
tacgatgttccagattacgct**atggagctggtgccctctgcccgtgcggagctgcag**
tcctcgcccctcgtcaacctctcggacgcctttcccagcgccttccccagcgcgggc
gccaatgcgtcggggtcgccgggagcccgtagtgcctcgtccctcgccctagccatc
gccatcaccgcgctctactcggctgtgtgcgcagtggggcttctgggcaacgtgctc
gtcatgtttggcatcgtccggtgagtcagccgagggcgtcgcggggtttggtcccag

ggtcagagtggactctactctttgtagaccaggatacccaaccctgactccccgggt
tcctgaatgtcctgcacagaggccaggtcctctgagattatgaggacaaggcgtgtg
tgtgtgtgtgtgtgtgtgtgtgtgtgtgtgcttgcactagcctaactacataacact
taatcatccttgacatctggtacccggggatcctctagagtcgacgaagttcctatt
ctctagaaagtataggaacttctatacgcgcccccagctggttctttccgcctcaga
agccatagagcccaccgcatccccagcatgcctgctattgtcttcccaatcctcccc
cttgctgtcctgccccaccccacccccccagaatagaatgacacctactcagacaatg
cgatgcaatttcctcattttattaggaaaggacagtgggagtggcaccttccagggt
caaggaaggcacgggggaggggcaaacaacagatggctggcaactagaaggcacagt
cgaggctgatcagcgagctctagagaattgatccccacgcgtcctaggtcagtacag
ctcgtccatgccgagagtgatcccggcggcggtcacgaactccagcaggaccatgtg
atcgcgcttctcgttggggtctttgctcagggcggactgggtgctcaggtagtggtt
gtcgggcagcagcacggggccgtcgccgatgggggtgttctgctggtagtggtcggc
gagctgcacgctgccgtcctcgatgttgtggcggatcttgaagttcaccttgatgcc
gttcttctgcttgtcggccatgatatagacgttgtggctgttgtagttgtactccag
cttgtgccccaggatgttgccgtcctccttgaagtcgatgcccttcagctcgatgcg
gttcaccagggtgtcgccctcgaacttcacctcggcgcgggtcttgtagttgccgtc
gtccttgaagaagatggtgcgctcctggacgtagccttcgggcatggcggacttgaa
gaagtcgtgctgcttcatgtggtcggggtagcggctgaagcactgcacgccgtaggt
cagggtggtcacgagggtgggccagggcacgggcagcttgccggtggtgcagatgaa
cttcagggtcagcttgccgtaggtggcatcgccctcgccctcgccggacacgctgaa
cttgtggccgtttacgtcgccgtccagctcgaccaggatgggcaccaccccggtgaa
cagctcctcgcccttgctcaccatatgcatgggccccttaaggctagctgggccagg
attctcctcgacgtcaccgcatgttagcagacttcctctgccctccatggcaccggg
cttgcgggtcatgcaccaggtgcgcggtccttcgggcacctcgacgtcggcggtgac
ggtgaagccgagccgctcgtagaaggggaggttgcggggcgcggaggtctccaggaa
ggcgggcaccccggcgcgctcggccgcctccactccggggagcacgacggcgctgcc
cagacccttgccctggtggtcgggcgagacgccgacggtggccaggaaccacgcggg
ctccttgggccggtgcggcgccaggaggccttccatctgttgctgcgcggccagccg
ggaaccgctcaactcggccatgcgcgggccgatctcggcgaacaccgcccccgcttc
gacgctctccggcgtggtccagaccgccaccgcggcgccgtcgtccgcgacccacac
cttgccgatgtcgagcccgacgcgcgtgaggaagagttcttgcagctcggtgacccg
ctcgatgtggcggtccggatcgacggtgtggcgcgtggcggggtagtcggcgaacgc
ggcggcgagggtgcgtacggccctggggacgtcgtcgcgggtggcgaggcgcaccgt
gggcttgtactcggtcatattggctgcaggtcgaaaggcccggagatgaggaagagg
agaacagcgcggcagacgtgcgctttgaagcgtgcagaatgccgggcctccggagg
accttcgggcgcccgccccgcccctgagcccgcccctgagcccgcccccggacccac
cccttcccagcctctgagcccagaaagcgaaggagcaaagctgctattggccgctgc
cccaaaggcctacccgcttccattgctcagcggtgctgtccatctgcacgagactag
tgagacgtgctacttccatttgtcacgtcctgcacgacgcgagctgcggggcggggg
ggaacttcctgactaggggaggagtagaaggtggcgcgaagggccaccaaagaacg
gagccggttggcgcctaccggtggatgtggaatgtgtgcgaggccagaggccacttg
tgtagcgccaagtgcccagcggggctgctaaagcgcatgctccagactgccttggga
aaagcgcctcccctacccggtagggcgcgtataactgaagttcctattctctagaaa

gtataggaacttcacgcgtgagagagacgcgtataacttcgtatagcatacattata
cgaagttataagctt(gggacagtttggcaacactttgtgtgtctgccgcgcacgca
gtttgtgattgggcagtttgacggtagcgtgtgtgttcgatcctctgaaaccttgca
agggtgagctgaggtgctctacaaaataccttaggtctttggagcatacaacaggga
cagactgtctgctccgggctgtggggacacgccccaagtcacatttagtagtcggtc
ttctgtggttgctgtgtgtgtgcttgtcccaagccatcagtttgtaattctgtatga
gactgagagtgtatttgcattgtttcttgtcaaacctctaggtcataggtgatggag
gcacccgggatgactttgaacaccagtctttgtgctgcgtgttctctgatggagagc
cccgtagttcctgtgaactggccatggggttctctcttcccttccccagtggccatg
tggtcaggaactcgggatatgattcagggcagatttatcctccttgaggcctgggct
acagaatgaacttaaggccagctagagaagagagagagggagggagggagggaggga
gggagggagagagagagagagagagagagagagagagagagagagagagagagatta
ttagtaagggctttaggggccacagactgtacgcttaattgatagcttctgcagcat
gtgtgaatccttacattagtttgtagtgttcagtccccagcacactacacactggac
atggtggtcccacggctgtaatcccagtcctgggggtgttgaagcagaaggctcag
aagttcaaagtcatgctttggttacatagagaatttgaggccagactaggataatgg
gcttggggtatagctaaatggcagggtacttgcctccagacaggcaaggctgagaac
tgcctaaattaacaaagcaaaatgtctccaacccatctgtacctgtgggcgcactga
tgtaagtgaagcacccctgcagaccccagaccgaggtaagggctcatagcaggctc
cacaatatgcaataagcaaacgagaggttttgctttcctctctgtttgctgccaagg
cgccaggggctgctgagatgagttttgagatttgggagcactaagaccctgtcttgt
ttagacagcatgtgtctcttgcctctgtcccctctcttgctcagattaggggttcag
)gcggccgcttcgaggccggtctccctatagtgagtcgtattaatttcgataagcca
ggttaacctgcattaatgaatcggccaacgcgcggggagaggcggtttgcgtattgg
gcgctcttccgcttcctcgctcactgactcgctgcgctcggtcgttcggctgcggcg
agcggtatcagctcactcaaaggcggtaatacggttatccacagaatcaggggataa
cgcaggaaagaacatgtgagcaaaaggccagcaaaaggccaggaaccgtaaaaaggc
cgcgttgctggcgtttttccataggctccgcccccctgacgagcatcacaaaaatcg
acgctcaagtcagaggtggcgaaacccgacaggactataaagataccaggcgtttcc
ccctggaagctccctcgtgcgctctcctgttccgaccctgccgcttaccggatacct
gtccgcctttctcccttcgggaagcgtggcgctttctcaatgctcacgctgtaggta
tctcagttcggtgtaggtcgttcgctccaagctgggctgtgtgcacgaaccccccgt
tcagcccgaccgctgcgccttatccggtaactatcgtcttgagtccaacccggtaag
acacgacttatcgccactggcagcagccactggtaacaggattagcagagcgaggta
tgtaggcggtgctacagagttcttgaagtggtggcctaactacggctacactagaag
gacagtatttggtatctgcgctctgctgaagccagttaccttcggaaaaagagttgg
tagctcttgatccggcaaacaaaccaccgctggtagcggtggtttttttgtttgcaa
gcagcagattacgcgcagaaaaaaaggatctcaagaagatcctttgatcttttctac
ggggtctgacgctcagtggaacgaaaactcacgttaagggattttggtcatgagatt
atcaaaaaggatcttcacctagatccttttaaattaaaaatgaagttttaaatcaat
ctaaagtatatatgagtaaacttggtctgacagttaccaatgcttaatcagtgaggc
acctatctcagcgatctgtctatttcgttcatccatagttgcctgactccccgtcgt
gtagataactacgatacgggagggcttaccatctggccccagtgctgcaatgatacc
gcgagacccacgctcaccggctccagatttatcagcaataaaccagccagccggaag

ggccgagcgcagaagtggtcctgcaactttatccgcctccatccagtctattaattg
ttgccgggaagctagagtaagtagttcgccagttaatagtttgcgcaacgttgttgc
cattgctacaggcatcgtggtgtcacgctcgtcgtttggtatggcttcattcagctc
cggttcccaacgatcaaggcgagttacatgatcccccatgttgtgcaaaaaagcggt
tagctccttcggtcctccgatcgttgtcagaagtaagttggccgcagtgttatcact
catggttatggcagcactgcataattctcttactgtcatgccatccgtaagatgctt
ttctgtgactggtgagtactcaaccaagtcattctgagaatagtgtatgcggcgacc
gagttgctcttgcccggcgtcaatacgggataataccgcgccacatagcagaacttt
aaaagtgctcatcattggaaaacgttcttcggggcgaaaactctcaaggatcttacc
gctgttgagatccagttcgatgtaacccactcgtgcacccaactgatcttcagcatc
ttttactttcaccagcgtttctgggtgagcaaaaacaggaaggcaaaatgccgcaaa
aaagggaataagggcgacacggaaatgttgaatactcatactcttcctttttcaata
ttattgaagcatttatcagggttattgtctcatgagcggatacatatttgaatgtat
ttagaaaaataaacaaataggggttccgcgcacatttccccgaaaagtgccacctga
cgtctaagaaaccattattatcatgacattaacctataaaaataggcgtatcacgag
gccctttcgtctcgcgcgtttcggtgatgacggtgaaaacctctgacacatgcagct
cccggagacggtcacagcttgtctgtaagcggatgccgggagcagacaagcccgtca
gggcgcgtcagcgggtgttggcgggtgtcggggctggcttaactatgcggcatcaga
gcagattgtactgagagtgcaccatatggacatattgtcgttagaacgcggctacaa
ttaatacataaccttatgtatcatacacatacgatttaggtgacactata
